# Into the Square and out of the Box: The effects of Quadrato Motor Training on Creativity and Alpha Coherence

**DOI:** 10.1371/journal.pone.0055023

**Published:** 2013-01-30

**Authors:** Tal Dotan Ben-Soussan, Joseph Glicksohn, Abraham Goldstein, Aviva Berkovich-Ohana, Opher Donchin

**Affiliations:** 1 The Leslie and Susan Gonda (Goldschmied) Multidisciplinary Brain Research Center, Bar-Ilan University, Ramat Gan, Israel; 2 Department of Criminology, Bar-Ilan University, Ramat Gan, Israel; 3 Department of Psychology, Bar-Ilan University, Ramat Gan, Israel; 4 Department of Neurobiology, Weizmann Institute of Science, Rehovot, Israel; 5 Department of Biomedical Engineering, Ben-Gurion University, Be’er-Sheva, Israel; Catholic University of Sacro Cuore, Italy

## Abstract

The objective of the present study was to investigate the body-cognitive relationship through behavioral and electrophysiological measures in an attempt to uncover the underlying mediating neuronal mechanism for movement-induced cognitive change. To this end we examined the effects of *Quadrato Motor Training* (QMT), a new whole-body training paradigm on cognitive performance, including creativity and reaction time tasks, and electrophysiological change, using a within-subject pre-post design. Creativity was studied by means of the Alternate Uses Task, measuring ideational fluency and ideational flexibility. Electrophysiological effects were measured in terms of alpha power and coherence. In order to determine whether training-induced changes were driven by the cognitive or the motor aspects of the training, we used two control groups: *Verbal Training* (VT, identical cognitive training with verbal response) and *Simple Motor Training* (SMT, similar motor training with reduced choice requirements). Twenty-seven participants were randomly assigned to one of the groups. Following QMT, we found enhanced inter-hemispheric and intra-hemispheric alpha coherence, and increased ideational flexibility, which was not the case for either the SMT or VT groups. These findings indicate that it is the combination of the motor and cognitive aspects embedded in the QMT which is important for increasing ideational flexibility and alpha coherence.

## Introduction

Philosophers, psychologists, and neuroscientists who study embodied cognition propose that cognition, ranging from high level mental constructs, such as creativity, to performance on various cognitive tasks (e.g. reaction time), is shaped by the body (motor and sensory systems), and its interactions with the environment [1]-[3]. The aim of the current work was to investigate the body-cognitive relationship through behavioral and electrophysiological measures in a preliminary attempt to uncover the underlying mediating neuronal mechanism for movement-induced cognitive change.

### 1.1 The Embodiment of Cognition

The embodied cognition hypothesis suggests that the neural networks related to cognition, and more specifically to conceptual thought and creative problem solving, are closely related to perception and action. It further argues that concepts arise from sensory-motor systems activation, which in turn form the building blocks of abstract and creative thought (for reviews see [4] and [5]). More broadly, it relies on a body of literature emphasizing the embodiment of consciousness [6]-[9]. Diverse neuroscientific fields support the embodied cognition hypothesis. An important illustration comes from the learning disabilities field. For example, people with Attention Deficit Disorder, in addition to the cognitive deficits, manifest motor deficits, such as grasping difficulties as well as slow and inaccurate responses [10], [11]. Additional support comes from neurodegenerative diseases. For example, Parkinson patients, in parallel to the severe motor deficits, suffer from cognitive decline and decreased mental flexibility [12], [13].

### 1.2 Whole-body Training and Electrophysiological Measures

Electrophysiological studies investigating whole-body training induced change (for review see [14]) have almost exclusively concentrated on power analyses, and generally reported increased frontal alpha (8–13 Hz) power (for review see [15]). These results do not seem to be linked to aerobic exercise, since also low-impact, whole-body training showed significant effects on cognitive performance. For example, both Yoga and Tai Chi have been noted to increase frontal EEG alpha power [16], [17]. Despite these interesting findings, the underlying mechanisms relating to whole-body training and cognition are not well understood.

The two measures of EEG activity which govern the literature are power and various measures for functional connectivity, the most frequent being coherence. EEG power is a gross measure for the summation of synchronous activity of thousands to millions of neurons in the underlying cortex. Functional connectivity, on the other hand, is a dynamical interaction characterized by synchrony relationships between the activities of two neuronal populations [9]. While numerous studies related to mental training and motor learning report changes in both alpha power and coherence, the relationship between both has not yet been fully elucidated. For example, a positive relationship has been reported following Transcendental Meditation (TM) training, where practitioners exhibit both higher frontal alpha power and coherence, compared to controls [18], [19]. Yet, other studies find no connection or a mixed relationship. For example, the effect of cognitive effort in desynchronizing alpha translated into mixed effects on coherence [20], and movement tasks increase alpha coherence and decrease power [21], [22]. Therefore, the exact effect of whole-body training on electrophysiology, especially coherence, is currently unknown.

### 1.3 Possible Measures of Cognitive Function

In order to examine cognitive change as a result of body-based training, most researchers have used tasks that test lower cognitive functions, such as reaction time (for reviews see [23] and [24]); while only a few examined creativity [25], [26]. Although reaction time (RT) is one of the most widely used measures of cognitive performance, it is not sufficiently sensitive to evaluate changes in other cognitive functions, such as creativity [24] that may occur as a result of mental and motor training. Creativity is an important aspect of cognition through which we can cope with significant challenges in our environments in novel and appropriate ways [27], [28]. A main feature of creativity is *ideational flexibility*, defined as the tendency to generate a heterogeneous pool of responses, or to use a variety of categories and themes when producing ideas [29]. Ideational flexibility conveys information that is not conveyed by ideational fluency, defined as the total number of ideas generated [29], [30].

Regarding the relationship between electrophysiology and cognition, findings demonstrate a connection between frontal alpha power and performance in different cognitive tasks, such as reaction time and creativity tasks [22], [26]. Furthermore, inter-hemispheric and intra-hemispheric alpha coherence are essential for cognitive functions. For example, both frontal and fronto-parietal alpha coherence were found to be related not only to cognitive functions, but also to motor functions, such as upper limb movements [31]-[34].

### 1.4 The Study Questions and Hypotheses

The main aim of the current exploratory work we present here was to study the link between whole-body training and cognitive as well as electrophysiological changes. Three general research questions guided our study: 1) what are the cognitive effects of whole-body training?; 2) what are the electrophysiological effects of the training?; and 3) are the cognitive and electrophysiological training-induced effects related?

To this end, we introduce here a new whole-body training paradigm, *Quadrato Motor Training*. Quadrato Motor Training (QMT) is a training paradigm developed by Patrizio Paoletti, as part of an education model [35]. The QMT is whole-body movement training with oral instructions, aimed at increasing attention, coordination and creativity. Using a pre-post design, we examined alpha power and inter-hemispheric and intra-hemispheric coherence. We concentrated on alpha activity due to the abundant literature connecting it both to the cognitive and motor neuroscientific domains [22], [26], [31]-[34], [36]-[37]. In addition, we measured cognitive performance, operationalized by two tasks measuring creativity and reaction time. Creativity was measured using the Alternate Uses (AU) task [38], which has been previously used to study changes in ideational flexibility following whole-body training [39]. Thus, we measured changes in cognitive performance following QMT, while attempting to relate it to possible changes in alpha band activity.

In order to determine whether training-induced changes were driven by the cognitive or the motor aspects of the QMT, we used two control groups: 1) *Verbal Training* (VT) - identical cognitive training with verbal response - this was meant to control for the motor load while keeping the same cognitive load and uncertainty, by instructing participants to only make verbal responses; and 2) *Simple Motor Training* (SMT) - similar motor training with reduced choice requirements, or the number of optional directions of movement at each choice point - this was meant to control for the cognitive load by reducing task complexity and uncertainty, while keeping the same motor load.

Specifically, we made the following three predictions regarding the QMT, as opposed to both SMT and VT: (1) QMT will enhance creativity and decrease reaction time; (2) QMT will increase frontal alpha power, interhemispheric and intrahemispheric coherence; (3) Increased creativity and faster reaction time will be correlated with increased frontal alpha power and coherence.

## Methods

### 2.1. Participants and Design

A total of 27 female students (20–35 years, SD = 2.5) participated in the study. All were right-handed with no medical history that might affect their EEG. We chose to focus in this study on females - as performance in different cognitive tasks, such as creativity, as well as their electrophysiological correlates, are both known to be gender-dependent [40]-[42]. The study was approved by the ethics committee of Bar-Ilan University, where the research took place.

Upon entering the lab, the participant signed a written informed consent. Subsequently, participants were seated in a quiet room, in front of a computer screen and completed the AU task. Then, we recorded baseline EEG for 5 minutes (2.5 minutes eyes open and fixed and then 2.5 minutes eyes closed). Subsequently, the RT Task was presented. The participants also completed a time production task and an embedded figures task reported elsewhere [43], [44].

All data were collected both before and after a single training session lasting 7 minutes in each of the three experimental training groups, with 9 participants randomly allocated to each group: (1) Quadrato Motor training (QMT- 3 choices and whole-body response); (2) Simple motor training (SMT - 1 choice and whole-body response); and (3) Verbal training (VT - 3 choices and verbal response).

### 2.2 Training Groups

#### 2.2.1 Quadrato Motor Training (QMT)

**Figure 1:**
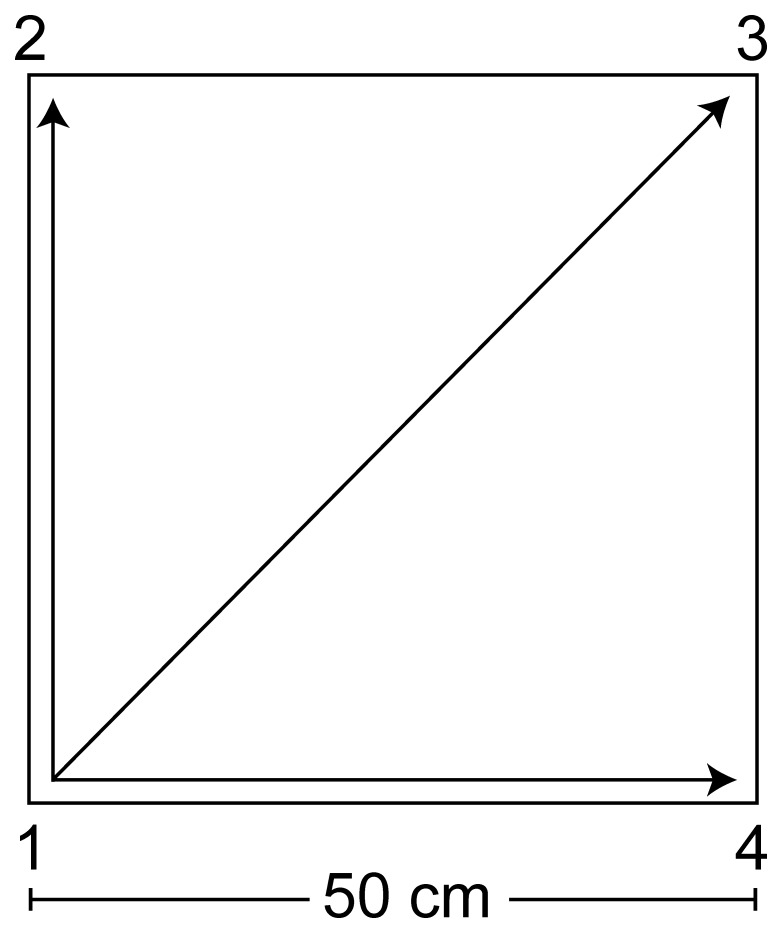
Graphical illustration of the Quadrato Motor training.

The Quadrato motor training group practiced the QMT in full. The QMT [44], [45] requires standing at one corner of a 0.5 m × 0.5 m square and making movements to different corners of the square in response to verbal instructions given by an audio tape recording indicating the next corner to which the participant should move (“one four” means move from corner 1 to corner 4). There are 3 optional directions of movement, and the movement is always in one step. We used a specific sequence of movements provided by Patrizio Paoletti, founder of the QMT training, translated from Italian to Hebrew by the first author. Each movement can be forward, backward, left, right, or diagonal. The instructions direct participants to keep the eyes focused straight ahead, hands loose at the side of the body. They are also told to immediately continue with the next instruction and not to stop due to mistakes. At each corner, there are three possible directions to move (for example, from corner 1 the participant can move to corner 2, to corner 3 or to corner 4). The training thus consists of 12 possible movements (3 directions × 4 corners): 2 forward, 2 backward, 2 left, 2 right and 4 diagonals. The participant is required to move from one corner to another according to the number on the recording. For example, if the sequence required is 1, 2, 1, 2, 1, 2, 3, 2, 4, 3, 1…. this means moving to the first corner, then to the second, then back to the first, and so on. See Figure 1.

Two important variables that were addressed in other contexts of motor learning are limb velocity required in order to accurately reach a goal and the decision regarding the responding limb [46], [47]. In the current study we aimed at controlling limb velocity, by using a movement sequence comprising a total of 69 instruction steps, paced at a rate of 0.5 Hz (similar to a slow walking rate), which was the same for all participants. We also controlled for the decision regarding the responding limb by instructing participants to begin all movements with the leg closest to the center of the square.

#### 2.2.2 Simple Motor Training (SMT)

The Simple Motor training (SMT) group moved from corner to corner on the square in exactly the same manner as the QMT group, but their sequence was consistently 1-2-3-4-1 etc. While participants in the QMT group had three possible directions of movement from each location before the specific instruction required the actualization of one specific movement, thus a high degree of uncertainty, the SMT group had no uncertainty. Their movements, however, were very similar to those of the QMT subjects (The SMT consisted of 4 possible movements, whereas the QMT also made diagonal movements. In all other respects, including speed and tempo, their movements were identical to those of the QMT group). The SMT group thus provided a control of similar motor performance but with reduced cognitive demands. This group also practiced with the same recordings as the QMT group. However, while the QMT group was told that each number represented a different corner of the square, the SMT group was told to simply begin at a certain corner and to continue to the next corner clockwise in response to the instructions. That is, regardless of the number specified on the tape, they always moved in the same sequence. Importantly, they were never instructed to associate the numbers with the corners in any way, so the numbers they heard did not represent a conflicting command and had a simple cue meaning for them.

#### 2.2.3 Verbal Training (VT)

The verbal training group stood 1m in front of the square, but did not move on the corners of it. Instead, their instructions were to respond to the taped commands verbally by stating what direction of movement would be required in order to reach the corner specified by the command. For a movement from corner 1 to corner 2, they were required to say “straight;” for a movement from corner 1 to corner 3, they were required to say “diagonal”. The following is a list of all possible combinations and the appropriate response: 1–2, 4-3, “straight”; 2-1, 3–4, “back”; 1–3, 4-2, 3-1, 2–4, “diagonal”; 1–4, 2–3, “right”; 4-1, 3-2, “ left”.

### 2.3 Electrophysiological Measurements

EEG data were recorded using a 65-channel geodesic sensor net (Electrical Geodesics Inc., Eugene, USA), sampled at 500 Hz and referenced to the vertex (Cz) with analog 0.1–200 Hz band-pass filtering. Impedance was usually kept under 40 kΩ, lower than the customary 50 kΩ with this system [48]. EEG signals showing eye movements or muscular artifacts were manually excluded, and bad channels were replaced using spatial interpolation [49]. The data were referenced offline to average reference. The first 32 non-overlapping, artifact-free epochs of 2.048 sec duration were extracted from each electrode for further analysis from the eyes closed resting state period, as previously reported [44], [45]. In two cases, due to noisy data, only the first 16 epochs were used. The power spectral distribution was computed by multitaper analysis which is advantageous in minimizing power leakage, especially in longer signals [50] and has been used in other studies assessing electrophysiological changes following training [51], [52]. The multitaper analysis (using time-bandwidth product of 5 and FFT length of 1024) was based on an internet open source (a Matlab software package - http://chronux.org/). The average spectral power was grouped into frequency bands and log-transformed for normalization. Subsequently, log power was averaged across the 32 (or 16) 2.048 sec epochs. Coherence values were calculated from the multitapered auto-spectrums for each electrode and from their pair-wise cross-spectra. Since coherence is not normally distributed, the coherence values were transformed using Fisher’s *z* transformation.

**Figure 2:**
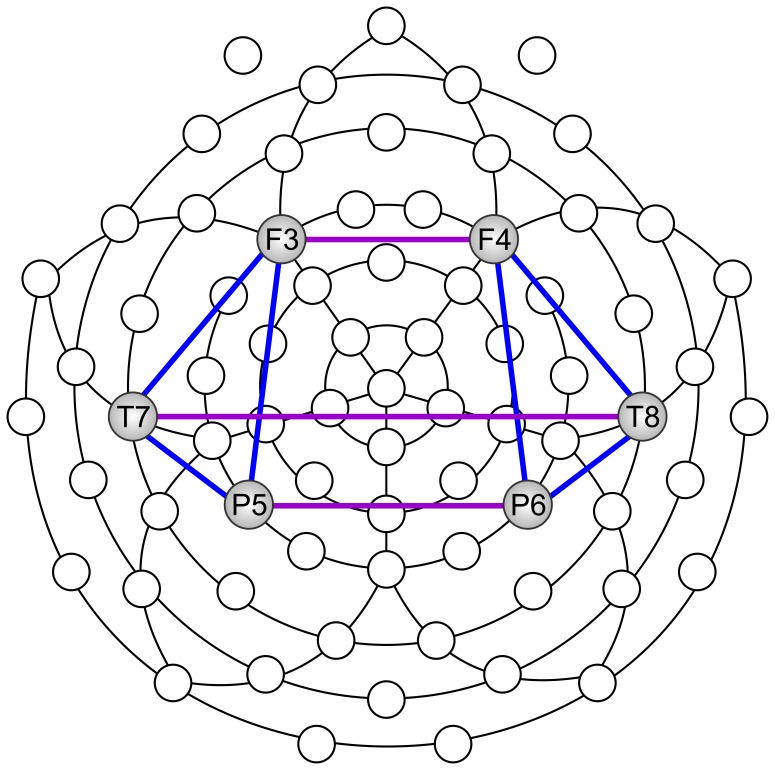
Scalp locations used to calculate EEG power and coherence. Scalp locations used to calculate power, as well as inter-hemispheric (purple) and intra-hemispheric (blue) coherences.

For the sake of data reduction and statistical comparisons, we chose one central electrode site in each region of interest. Since frontal, temporal and parietal areas are important for cognition and action [53], [54], we chose to focus on bilateral frontal, temporal and parietal electrode sites (F3, F4, T7, T8, P5, P6). For coherence analysis we defined electrode ‘pairs of interest’ (POI), on the basis of prior knowledge concerning hemispheric asymmetry and creativity [55], [56] - see Figure 2.

### 2.4 Cognitive Tasks

#### 2.4.1 Reaction Time (RT) task

The reaction time (RT) task consisted of 31 trials in which a flashing asterisk, 1 cm in height, appeared at the center of a computer screen for 150 ms and alerted the participant of an upcoming computer-generated tone (1200 Hz, 72 dB), with latency of 1500 ms. Participants were instructed to focus their eyes on the screen (50 cm distance) during the trial, and to end the tone as quickly as possible by pressing a button with the right index finger. The RT task was part of a contingent negative variation paradigm [19], whose electrophysiological results will be presented elsewhere. All reaction times were log-transformed to normalize the data.

#### 2.4.2 Alternate Uses task

The Alternate Uses (AU) task assesses creativity [36]. In this task, the participant is required to name as many different ways in which a given item might be used. For example, a shoe can be used to walk with or can serve as a drum. In a previous pilot study with the aim of examining changes in creativity following short term and long term training, we clustered a 908-word database developed by Levy-Drori and Henik [57], using hierarchical cluster analysis, and grouped those words having similar ratings of concreteness, availability of context and familiarity assigned by Levy-Drori and Henik. Concreteness was rated by them on a scale from one to seven, where “1” indicated very low concreteness (very abstract word) to “7” which indicated a very concrete word [58]. Availability of context was defined by Schwanenflugel and Shoben [59] as ranging from 1–7, with “1” indicating that it is very difficult to think of a context and “7” indicating that it is very easy. Familiarity was defined by Gilhooly and Logie [60] as ranging from “1” indicating that it is not very familiar and “7” indicating that it is very familiar. In the pilot study, we chose 18 words from the three largest clusters for which the level of concreteness was highest, and similarity on familiarity and availability features was highest (see Table 1). These 18 words were divided into two groups (9 words in each group). A total of 60 participants received one of the two lists, and were asked to produce as many alternate uses as possible, one minute being allocated for each item. Each word was shown on a single page on which the participant had to write down the various uses. The scores for each word were analyzed by counting the number of alternate uses produced. The words were then divided into 6 groups of 3 words so that each group had a similar number of alternate uses. In the main study, three items were successively shown on a computer screen before the training and three others were shown after the training. Presentation order of these items between groups was counterbalanced across subjects.

**Table 1:** Word list for the Alternate Uses task.

Word	Availability	Familiarity	Concreteness	Uses
Boot	4.67	4.22	6.56	4.17
Envelope	4.7	4.97	6.37	5.14
Bench	4.85	4.43	5.83	6
Screwdriver	4.6	3.86	6.09	4.83
Ladder	5	4.37	6.03	5.5
Drawer	4.54	4.29	6.29	4.67
Stand	4.83	4.17	5.94	3.67
Antenna	4.4	4.41	6.33	3.5
Tie	4.5	4.28	6.33	5.33
Shutter	4.65	4.66	6.37	5.5
Flowerpot	4.83	4.37	6.4	8
Box	4.5	4.22	6.3	7.5
Shelf	4.73	4.37	6.2	5.2
Umbrella	5.41	4.23	6.49	5.67
Scarf	4.43	4.13	6.52	6
Vase	4.43	3.97	6.56	5.5
Handkerchief	4.37	4.1	6.63	5.75
Pillow	4.67	4.97	6.52	5.75

Two main measures were computed from the AU task: Ideational fluency and ideational flexibility. The ideational fluency score was defined as the mean number of uses given by the participant for the three items [61]. On the basis of all the uses made by the participants, 10 independent categories were defined across all the items. These included broad categories of usage such as “a weapon” or “a costume.” The ideational flexibility score was defined as the mean number of different categories employed by the participant across all three words presented [62]. Hence, in order to calculate the flexibility score, all responses for a given item were first divided into the different independent categories. For example, using an item as a musical instrument and using it as a weapon were regarded as two independent categories; while using it as a drum and as a trumpet would be regarded as the same category. Since flexibility is a subjective measure it is important to examine its reliability [63]. Therefore, two independent judges who were naïve to the identity of the participants and their training groups scored the test independently for flexibility, and consistency between judges was tested. We examined the correlation between the scores of the two judges using a 2-tailed Pearson correlation coefficient test. A high correlation was found between scores given by the two judges both in the scores before the training and after the training: *r* = 0.89 and 0.87, *n* = 27, respectively.

Two other scores, thought to represent a failure to monitor responses [37] were also computed: the AU perseveration within object – measuring a repetition of the same use for one object (e.g., using a newspaper to wrap a book and using a newspaper to wrap a box of chocolates), and AU perseveration between objects – repeating the same use for different objects (e.g., throwing a shoe at somebody and throwing a pot at somebody).

### 2.5 Statistical Analyses

To answer the first research question regarding the cognitive effects following whole-body training, we ran a Group (QMT, SMT, VT) × Training (pre, post) analysis of variance (ANOVA) for creativity (separately, on ideational flexibility and ideational fluency scores) and on the log-transformed RTs. Preliminary inspection of the data revealed that for both ideational flexibility and ideational fluency, the post-pre difference scores had a symmetric distribution. Hence, no transformation of the data was required.

Then, in order to answer the second question concerning the electrophysiological effects of the training, we ran one ANOVA for log-transformed alpha power. This was a Group × Training × Hemisphere (left, right) × Electrode site (Frontal [F], Temporal [T] and Parietal [P]) ANOVA. Given that both inter-hemispheric and intra-hemispheric alpha coherence are important for cognitive and motor functions, we conducted two ANOVAs for alpha coherence. For the intra-hemispheric coherence we ran a 4-way ANOVA with Group × Training × Hemisphere × Electrode Pair (F-T, F-P, T-P) with repeated measures on the last three factors. For the inter-hemispheric coherence, we ran a 3-way ANOVA with Group × Training × Bilateral Electrode Pair (F-F,T-T, P-P). We adopted the Greenhouse-Geisser criterion for all ANOVAs reported in this paper. Whenever needed, we added post-hoc *t*-tests. In order to estimate the magnitude of the group differences, we computed effect sizes using Cohen’s *d* for the comparisons where significant differences were observed.

Turning to the third question related to the possible relation between the cognitive and electrophysiological training-induced effects, we used Pearson correlations. In order to avoid multiple comparisons, we focused only on frontal alpha power and coherence - which are also the most prominently reported in relation specifically to RT and creativity [22], [64].

## Results

### 3.1. Cognitive Measures

#### Alternate uses task

**Figure 3:**
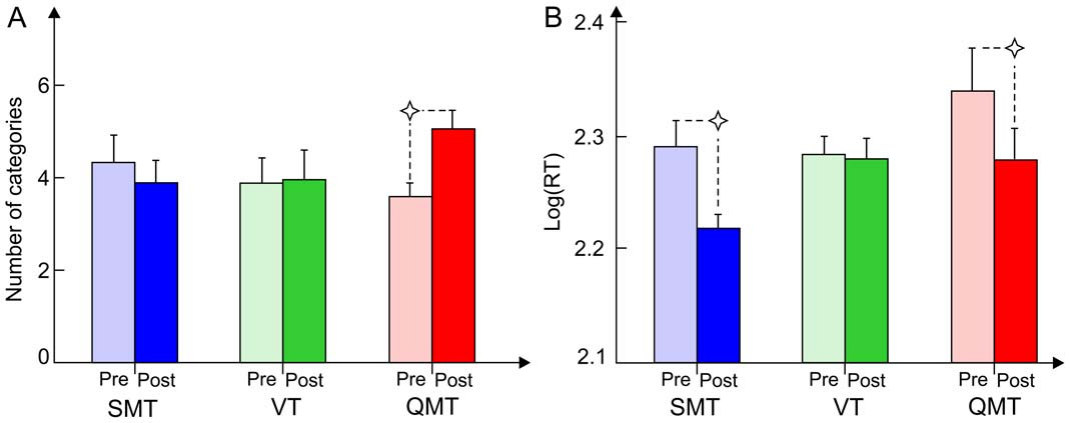
Cognitive change as a function of Group and Training. Figure 3A shows ideational flexibility as a function of Group and Training. Data are expressed as mean ± SEM*, *p*<0.01. Figure 3B shows log(RT) as a function of Group and Training. Data are expressed as mean ± SEM*, *p*<0.05.

The first ANOVA was designed to answer the question concerning the whole-body training-induced effects on ideational flexibility. This yielded a significant Group × Training interaction [*F*(2, 24) = 3.8, *MSE* = 10.58, *p*<0.05]. For QMT, ideational flexibility significantly increased (for pre-post scores, *t*(8) = −5.22, *p*<0.01, Cohen’s *d* = 1.24) in contrast to SMT and VT which showed no change following training (see Figure 3A). The second ANOVA which was conducted for ideational fluency did not reveal a main effect for Training nor a Group × Training interaction [*F*(1, 24) <1, *F*(2, 24) <1, respectively]. Neither main effect for Training nor a Group × Training interaction was found for the AU perseveration-within or between object indices.

#### Reaction time

The third ANOVA, designed to answer the question concerning the whole-body training-induced effects on reaction time, yielded a main effect for Training [*F*(1, 24) = 14.81, *MSE* = 0.002, *p*<0.01], showing faster RT following training. Although the Group × Training interaction was only marginally significant [*F*(2, 24) = 3.17, *MSE* = 0.002, *p* = 0.06], *t*-test analysis demonstrated that both motor groups (QMT and SMT) significantly improved in speed [*t*(8) = 3.26 and 2.99, *p*<0.05, Cohen’s *d* = 0.59 and 1.25, respectively], see Figure 3B.

### 3.2. Electrophysiological Data

**Figure 4:**
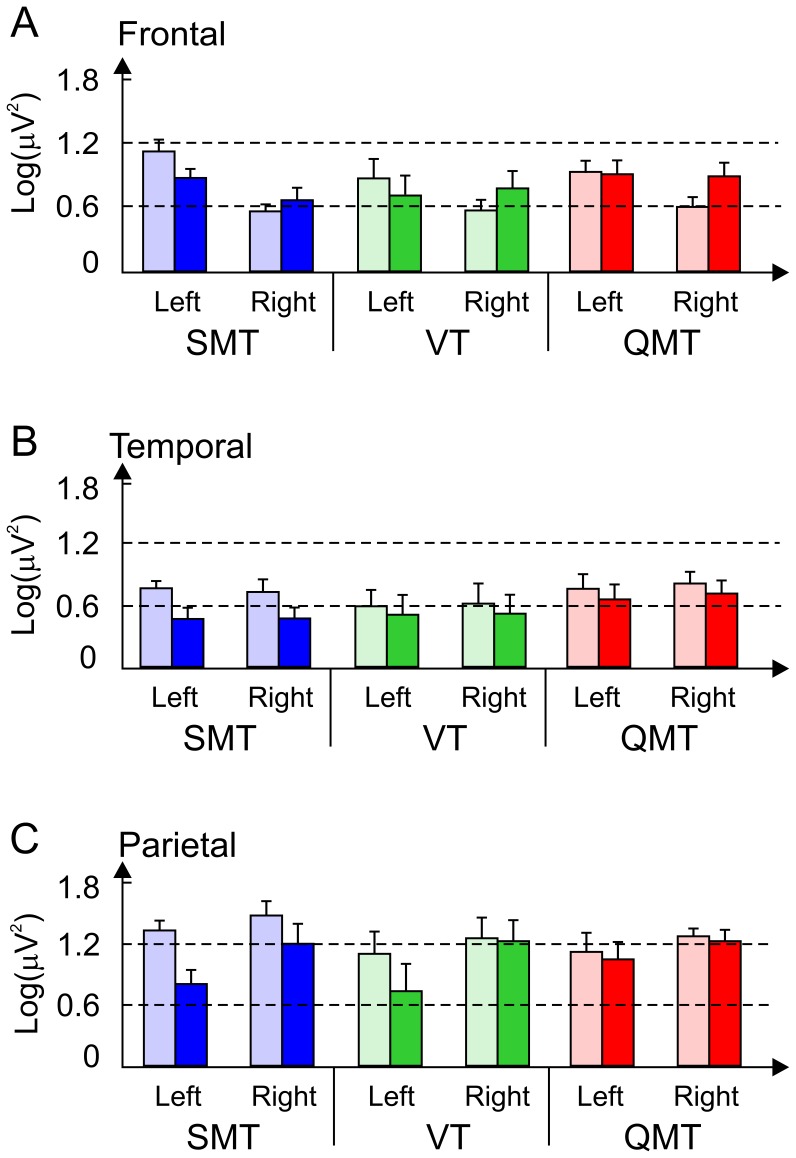
Alpha power as a function of Group, Hemisphere, Electrode and Training. The figure shows log-transformed alpha power as a function of Group, Hemisphere, Electrode and Training. Data are expressed as mean ± SEM.

The first ANOVA, designed to answer the question concerning the alpha power effects of the training, yielded a main effect for Training [*F*(1, 24) = 17.57, *MSE* = 0.149, *p*<0.001] as well as a Training × Hemisphere × Electrode site interaction [*F*(2, 48) = 4.12, *MSE* = 0.032, *p*<0.05]. As there were no group interactions, the specific changes within the groups are not specified. However as seen in Figure 4, the trend in power was a decrease in the left hemisphere (especially in the SMT group).

**Figure 5:**
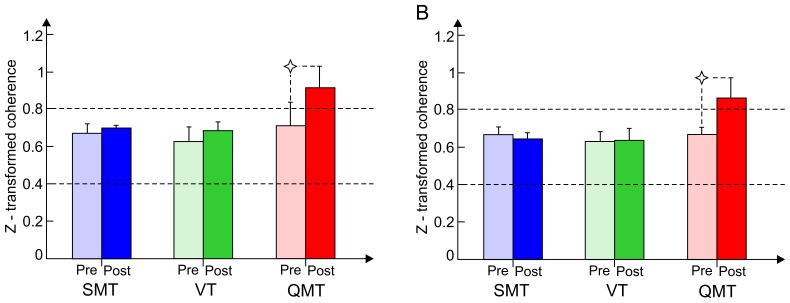
Alpha coherence as a function of Group and Training. Figure 5A shows intra-hemispheric *z*-transformed alpha coherence as a function of Group and Training. Intra-hemispheric alpha coherence was calculated for right and left fronto-temporal, fronto-parietal and temporo-parietal electrode sites together. Figure 5B shows mean inter-hemispheric *z*-transformed alpha coherence, which was calculated for bilateral frontal, temporal and parietal electrode sites. Data are expressed as mean ± SEM*, *p*<0.05.

The second ANOVA, testing the training-induced effect on *z*-transformed intra-hemispheric alpha coherence, yielded a significant Group × Training interaction [*F*(2, 24) = 3.82, *MSE* = 0.06, *p*<0.05]. As seen in Figure 5A, unlike the VT and SMT groups, only the QMT group demonstrated a significant increase in intra-hemispheric alpha coherence (*t*(8) = −2.68, *p*<0.05). Following the suggestion of a reviewer, we explored this increase in alpha coherence for the QMT. For 4 pairs (left F-T, left F-P, left T-P, right F-T), intra-hemispheric coherence increased (from.704,.705,.708, and.546, respectively, to 1.064,.865,.953, and.951, respectively). For the 2 other pairs (right F-P, right T-P), intra-hemispheric coherence remained the same (from.742 and.886, respectively, to.732 and.946, respectively). Clearly, then, the greatest degree of increase in intra-hemispheric coherence is in bilateral F-T (Δ = .36 and.41, respectively).

The third ANOVA, testing the training-induced effect on *z*-transformed inter-hemispheric alpha coherence, yielded a significant a Group × Training interaction [*F*(2, 24) = 3.87, *MSE* = 0.05, *p*<0.05]. As seen in Figure 5B, although only marginally significant, only the QMT group demonstrated increased inter-hemispheric alpha coherence (*t*(8) = −2.17, *p* = 0.06), similarly to intra-hemispheric coherence.

### 3.3. Correlations between Electrophysiology and Cognitive Change

We then investigated whether the increased ideational flexibility and faster RTs were positively correlated with the observed changes in bilateral frontal (F3–F4) alpha coherence, or frontal alpha left and right power, by a Pearson correlation. Change in ideational flexibility was calculated by the subtraction of the number of independent categories before training from the number of categories after training. Change in Log(RT) was calculated by subtraction of post from pre training Log(RT), and change in coherence was calculated by the subtraction of pre from post training *z*-score coherence values. Due to multiple (a total of 6) tests, we report a significant correlation only when *p*<0.01.

**Figure 6:**
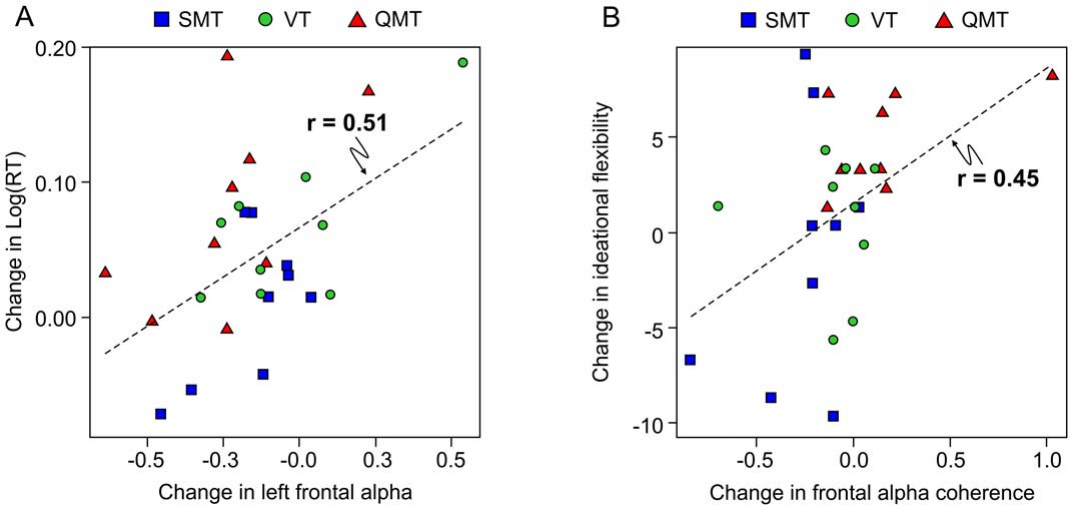
Correlations between change in frontal alpha activity and change in cognitive performance. Correlations between change in (A) frontal alpha power and log(RT) and (B) frontal alpha (F3–F4) alpha coherence and ideational flexibility.

As can be seen in Figure 6, change in left frontal alpha power was significantly and positively correlated with change in reaction time (*r* = 0.51, *p*<0.01, *n* = 27), while change in frontal alpha coherence was significantly correlated with change in ideational flexibility (*r* = 0.45, *p*<0.01, *n* = 27).

In line with the suggestion of a reviewer, in order to rule out the possibility that the significant correlation between change in frontal alpha coherence and change in ideational flexibility was due to one outlier in the QMT group, we reanalyzed the data without that participant, and found that change in frontal alpha coherence was still significantly correlated with change in ideational flexibility (*r* = 0.52, *p*<0.01, *n* = 26).

## Discussion

### 4.1 Whole-body Training and Cognition

We report here a training-induced change in cognitive performance. More specifically, we report faster RT following QMT and SMT, as well as increased ideational flexibility in the QMT group (Figure 3). While ideational flexibility increased following QMT, this was not the case in either the SMT group or in the VT group, representing the motor and cognitive aspects of the training, respectively. This is in line with our first hypothesis, suggesting improved creativity and RT following QMT. The current results are in agreement with previous findings of a positive effect of whole-body training on cognition [14]. Our results are consistent with the embodied-cognition hypothesis, claiming that neural networks related to cognition, and more specifically to conceptual thought and creative problem solving, are closely related to action [4], [5].

Why does QMT enhance ideational flexibility, while SMT or VT do not? We suggest that this stems from the complexity of the movement, a topic largely neglected thus far. Up till now there have not been direct references to the number of optional directions of movement and the degree of motor activity required in whole-body training. Noteworthy is the fact that information load during whole-body training may be different as a consequence of the degree of motor activity and thus the recruitment of motor units required. For example, dance may require recruitment of more motor units for the execution of the coordinated movement of arms and legs in comparison to walking [65]. In addition, more complex whole-body training, involving a greater number of optional directions of movement and/or a greater degree of motor activity may require greater information load. Greater information load, such as finger movement sequences of increasing complexity are known to recruit additional neuronal synchronization [66]. Thus, it is perhaps the combination of the cognitive and motor aspects that increases creativity. The difference between the results observed in the two motor groups in the creativity task, in contrast to the RT task, support previous claims that simple cognitive tests measuring RT are not sufficiently sensitive to evaluate changes in other cognitive functions that may occur following different kinds of training [24].

As a reviewer has suggested to us, another explanation for the specific improvement in ideational flexibility could be that QMT was a more engaging task than SMT and VT, thus allowing the participant to become bodily centered in a state of flow [67]. Flow is a state of high attention occurring during active performance, characterized by high creativity, as well as excellent emotional, cognitive and motor experience [67]-[69]. In fact, previous research has found that positive emotions are associated with enhanced divergent-thinking performance compared to negative emotions [70]. Furthermore, increased cognitive flexibility and creative thinking are associated with positive emotion, possibly reflecting a change in selective attention [71]. Additional supporting evidence, although indirect, comes from the fact that the electrophysiological correlate of flow and exercise is similar to that of creativity; namely increased frontal alpha activity [72], [73]. Indeed, we found a significant correlation between increased bilateral frontal alpha coherence and cognitive flexibility, as we discuss below.

### 4.2 Whole-body Training and Electrophysiology

Our central electrophysiological finding relates to the changes in coherence. Specifically, we found increased inter and intra-hemispheric alpha coherence following QMT, in contrast to the VT and SMT (Figure 5). This is in line with our hypothesis of increased alpha coherence following QMT. However, in opposition to our hypothesis, we did not find an effect related to power. Yet, we found a general decrease in left alpha power. This discrepancy may be resolved when taking into consideration that decreased frontal alpha power is related to motor planning occurring at the contralateral side to the movement [22]. In addition it may be related to the possibility that while power may be the most relevant measure for examining neuronal change in cognitive and motor task in males, coherence may be more suitable for examining neuronal change in females [21], [41].

The fact that it was specifically the QMT group which exhibited increased alpha coherence suggests that incorporating a greater degree of complexity, namely, combining whole-body motor response with greater number of directions of movement may be important for these changes to occur. This suggestion is supported by a study showing that finger movement sequences of increasing complexity were accompanied by increased alpha coherence in comparison to simpler sequences [66]. We suggest that the QMT participants probably had to pay more attention to both the motor and cognitive levels for producing the correct direction of movement to the next point in the Quadrato space, than those in the SMT (having similar motor performance with minimal cognitive demands), or the VT (having the same number of options as the QMT, but only a verbal response). Indeed, alpha coherence has been linked to attention [74], [75].

### 4.3 Training-induced Cognitive Improvement and Electrophysiological Changes

We report here that while change in reaction time was correlated with change in frontal alpha power; change in ideational flexibility was correlated with change in alpha coherence. While the trend in power was a decrease in the left hemisphere mostly for the SMT, change in reaction time was positively correlated with increase in left alpha power. As reported above, change in ideational fluency was related to change in frontal alpha coherence, irrespective of group (Figure 6), although the correlation was more evident for the QMT group. The correlation between the training-induced creativity, irrespective of group, with frontal alpha coherence may suggest that improved ideational flexibility following QMT may be mediated by increased alpha coherence. Due to the exploratory nature of the current report, more studies are needed to elucidate this important issue and new hypothesis. Research studying mental training in the form of various meditation techniques has examined frontal alpha coherence and has found it to increase [18], [76], in parallel to increased creativity [77]. Interestingly, semi-structured interviews, which we conducted with participants following QMT, have demonstrated consistent meditative-like effect of the QMT training, which could be comparable to other meditation effects, and especially to body-based meditations [78]. These mainly include increased body perception, sense of lucidity and increased attention.

### 4.4. Summary and Possible Implications

The general aim of this study was to study whole-body training-induced effects on cognitive performance, electrophysiology, and the relation between them. To this end, we examined the cognitive and electrophysiological effects of Quadrato Motor Training (QMT), a training paradigm designed to increase attention, coordination and creativity. If greater understanding is to be achieved regarding the beneficial effects of different types of whole-body training and the core features enabling cognitive change, the different aspects of the whole-body training should be examined. To this end, we compared the QMT with two other training groups, SMT (simple Motor Training) and VT (Verbal Training), controlling for cognitive and motor load, respectively.

We show that: 1) whole-body training induces cognitive improvement. Specifically, training decreases reaction time (RT) irrespective of cognitive load (seen both in SMT and QMT), and QMT increases ideational flexibility; 2) QMT increases alpha coherence; 3) a general trend of left hemisphere decrease in alpha power, regardless of training group and 4) cognitive performance is positively correlated with alpha activity. Specifically, change in RT is positively correlated with change in frontal alpha power, and change in ideational flexibility is positively correlated with change in frontal alpha coherence.

The effectiveness of the QMT, as shown in this report, together with the ease of learning the task and the minimal space it requires, turn this training to a possibly useful paradigm for adolescents in the classroom, or elderly people. Additional research should examine QMT effectiveness in increasing cognitive performance with different populations suffering from reduced alpha coherence and impaired cognitive performance, such as Alzheimer patients [79], or with populations with decreased motor and cognitive function, such as different learning disabilities [80].
